# Determination of volatile organic compounds by HS‐GC‐IMS to detect different stages of *Aspergillus flavus* infection in Xiang Ling walnut

**DOI:** 10.1002/fsn3.2229

**Published:** 2021-03-11

**Authors:** Shan Wang, Haizhen Mo, Dan Xu, Huiling Hu, Liangbin Hu, Liang Shuai, Hongbo Li

**Affiliations:** ^1^ School of Food and Biological Engineering Shaanxi University of Science and Technology Xi’an China; ^2^ Department of Horticulture and Landscape Architecture Henan Institute of Science and Technology Xinxiang China; ^3^ College of Food and Biological Engineering Hezhou University Hezhou China

**Keywords:** *A. flavus*, gas chromatography, headspace, ion mobility spectrometry, volatile organic compounds, walnut

## Abstract

The aim of this study was to evaluate the performance of volatile organic compounds (VOCs) for evolution monitoring and early detection of *Aspergillus flavus* (*A. flavus*) contamination in walnuts. We successfully applied headspace–gas chromatography–ion mobility spectrometry (HS‐GC‐IMS) to evaluate walnut VOC changes caused by *A. flavus* contamination. A total of 48 VOCs were identified in walnuts contaminated with *A. flavus*. After identification of VOCs, a heat map and principal component analysis (PCA) highlighted ethyl acetate‐D, 3‐methyl‐2‐butanol, and cyclohexanone as potential biomarkers specific to *A. flavus* contamination in walnuts. These results provided valid targets for the development of sensors to evaluate the early mold contamination in stored walnuts.

## Graphical abstract

The aim of this study was to evaluate the performance of volatile organic compounds (VOCs) for evolution monitoring and early detection of *Aspergillus flavus* (*A. flavus*) contamination in walnuts. These results provided valid targets for the development of sensors to evaluate the early mold contamination in stored walnuts.

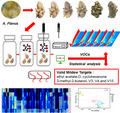

## 1 INTRODUCTION

*Aspergillus flavus*, a widely distributed saprophyte, is also an important soil fungus that produces highly carcinogenic aflatoxins (AFs) (Zhang et al., 2014). This fungus is also the most economically important in the *A. genus*, because it contaminates seed crops and foodstuffs with this toxic and carcinogenic secondary metabolite (Yang et al., 2016). *Aspergillus flavus,* as an important fungus, can cause damage to different plants, such as peanuts and maize, and produce the most hazardous AFs (Gonçalves et al., 2012; Moosavi Nasab et al., 2018; Noroozi et al., 2020). When AFs are present in foods at sufficiently high levels, these fungal metabolites can have toxic effects that range from acute (liver or kidney deterioration) to chronic (e.g., liver cancer) toxicity and can be mutagenic and teratogenic (Mahmoud et al., 2014). Thus, the ingestion of foods contaminated with aflatoxins poses a significant threat to human health due to its hepatotoxicity and immunotoxicity (Yang et al., 2015). In consideration of these detrimental properties, further research on the effect of *A. flavus* on food is important for developing effective strategies to control food safety in foodstuffs.

Walnuts (*Juglans regia* L.) are an extremely valuable nut species (antioxidant activity, and phenolic and mineral contents of the walnut kernel (*Juglans regia* L.) as a function of the pellicle color), and they contain the highest amount of PUFAs of edible nuts (Nakanishi et al., 2016). With the development of society, all people begin to pursue diversity, nutritional content, and safety to meet their dietary needs and food preferences for a positive and healthy life (Udomkun et al., 2018). So, walnuts are commonly found in the human diet because of their rich nutrients (Miao et al., 2020; Sánchez‐González et al., 2016). Unfortunately, contamination of walnuts by AFs produced by the fungi *A. *spp. is a serious problem because of their potential threat to health (Amini & Ghoranneviss, 2016). Some studies have investigated the implications of kernel oxidation and fungal growth (*A. *spp.) that result in the development of carcinogenic aflatoxins in relation to the commercial storage and transportation of walnuts (Campbell et al., 2003). Molds individuals are tiny and hard to detect in the early stages of growth. When the quality of the walnuts is changed to an abnormal state, the damage is irreparable. The inhibition of fungi before the toxins are produced is more important and is a better strategy than the removal of toxins once produced (Amini & Ghoranneviss, 2016). Therefore, there is an urgent need for a method that can accurately determine the extent of mold growth on walnuts and control this crisis early on. The occurrence of harmful compounds in foodstuffs can result from their mishandling during food production or can be formed during food production, processing, or storage (Hernández‐Mesa et al., 2017). Some harmful compounds are also produced in the process of AF contamination in walnuts. Flavor usually determines the overall unique sensory characteristics of food and is also an important tool for evaluating the nutritional value and freshness of food.

The conventional physical and chemical analysis methods for mold detection cannot achieve the requirements of fast and nondestructive testing because of their complex operation steps, time consumption, and poor sensitivity. With the development of chromatographic and spectral technology, the identification of mold in the food industry has started to turn to the detection of substances produced by the growth and metabolism of molds, such as mycotoxins and volatile organic compound biomarkers. Ion mobility spectrometry (IMS) is an instrumental analytical technique of separating the ions of detected substances based on their ion mobility velocity under atmospheric pressure (Zhang et al., 2016). Headspace–gas chromatography–ion mobility spectrometry (HS‐GC‐IMS) is a simple, rapid, and sensitive detection technique (Rodríguez‐Maecker et al., 2017). This instrumentation combines the outstanding separation capacity of gas chromatography with the advantages of fast response and high sensitivity of ion mobility spectrometry (Gerhardt et al., 2017). This technique has little requirement for sample pretreatment to identify volatile substances in liquid or solid samples (Cavanna et al., 2018). Over the few decades, this technique has been applied in many different research fields for the detection of chemical warfare agents, for security purposes, and for food quality and safety as well as for medical purposes (Jünger et al., 2010). In particular, it has been used for the detection of food‐borne microbial spoilers since spoilage of food is often accompanied by the formation of characteristic volatile compounds (Karpas et al., 2002). As a result, the HS‐GC‐IMS technique can separate and identify volatile compounds in complex matrices, such as aldehydes, ketones, alcohols, amines, and other volatiles. Considering these factors, HS‐GC‐IMS technology was used to establish an effective method to identify aroma compounds in walnut samples contaminated with *A. flavus* at different growth stages. Characteristic fingerprint spectra and heat map were used to characterize the infection process of *A. flavus*, and PCA with cluster analysis was used to explore the utilization of this method for the rapid assessment of the degree of walnut mildew and the feasibility of early warning of the degree of walnut mildew.

## 2 MATERIALS AND METHODS

### 2.1 Materials, fungal strains, and inoculum preparation

Unshelled butterfly walnuts (*Juglans regia L*., Xiangling Variety) were obtained from store for this study and preserved in high barrier bags at −20℃. *Aspergillus flavus* were a laboratory standard strain *NRRL3357*, purchased from China General Microbial Culture Collection Centre. This strain forms high concentrations of aflatoxin after growth on YES agar (20 g/L yeast extract, 150 g/L sucrose, 15 g/L agar) at 30°C for 4 days.

### 2.2 Pretreatment of walnut samples

First, the randomly selected walnuts were peeled and disinfected with 1% sodium hypochlorite. After washing the samples three times with sterile water, the surface of the samples was dried with sterilized filter paper. Then, walnut pulps of the same size, no pests, no mechanical damage were grouped and weighed about 5.5 g per group.

### 2.3 Preparation of mildew samples

The samples of the treatment groups were inoculated with a concentration of 10^6^/ml *A. flavus* spore suspension and placed on the water agar medium. Then, they were dried at room temperature and cultured in a 30°C incubator with constant temperature and humidity, and samples not inoculated with *A. flavus* spores were used as control. At the same time, each group of samples was set up with three parallel groups, a total of 18 groups of samples. Next, the sample changes were closely observed and sampled in a freezer at irregular intervals.

### 2.4 Testing conditions

A HS‐GC‐IMS Flavor Analyzer (FlavourSpec^®^) was used to identify volatile compounds in walnuts stored under different storage conditions. The gas chromatographic preseparation was performed at 45°C on a FS‐SE‐54‐CB‐0.5 capillary chromatographic column (15 m × 0.53 mm). Headspace incubation temperature, incubation time, and incubation speed were set at 60°C, 10 min, and 500 rpm, respectively. Nitrogen was used as carrier gas under the following programmed flow: 2 ml/min for 2 min, 100 ml/min at 20 min, and maintained for 10 min. The headspace injection needle temperature was 65°C, and the injection volume was 500 μL. MS parameters were as follows: full‐scan mode with scan range of 33–500 amu at a rate of 0.50 scan/s. The ion source temperature was 260°C with an ionizing energy of 70 eV and a mass transfer line of 250°C (Taylor et al., 2017).

### 2.5 Statistical analysis

The instrumental analysis software includes LAV (Laboratory Analytical Viewer) and three plug‐ins as well as GC × IMS Library Search, which can be used for sample analysis from different angles. The VOC identification was achieved by the National Institute of Standards and Technology (NIST) reference library (NIST Mass Spectral Library, version 2.0a, 2001) and the comparison of the retention times and mass spectra of authentic standards (Taylor et al., 2017). The spectra were analyzed using the LAV software, and the different profiles and fingerprints of volatile components were constructed using the Reporter and Gallery plug‐ins. The PCA and heat map were used for clustering analysis of walnut samples (Yang et al., 2019). The heat map and PCA were generated using the R software packages, pheatmap for heat maps, and factoextra for the PCA plots.

## 3 RESULTS

### 3.1 HS‐GC‐IMS analysis of walnut mold

**FIGURE 1:**
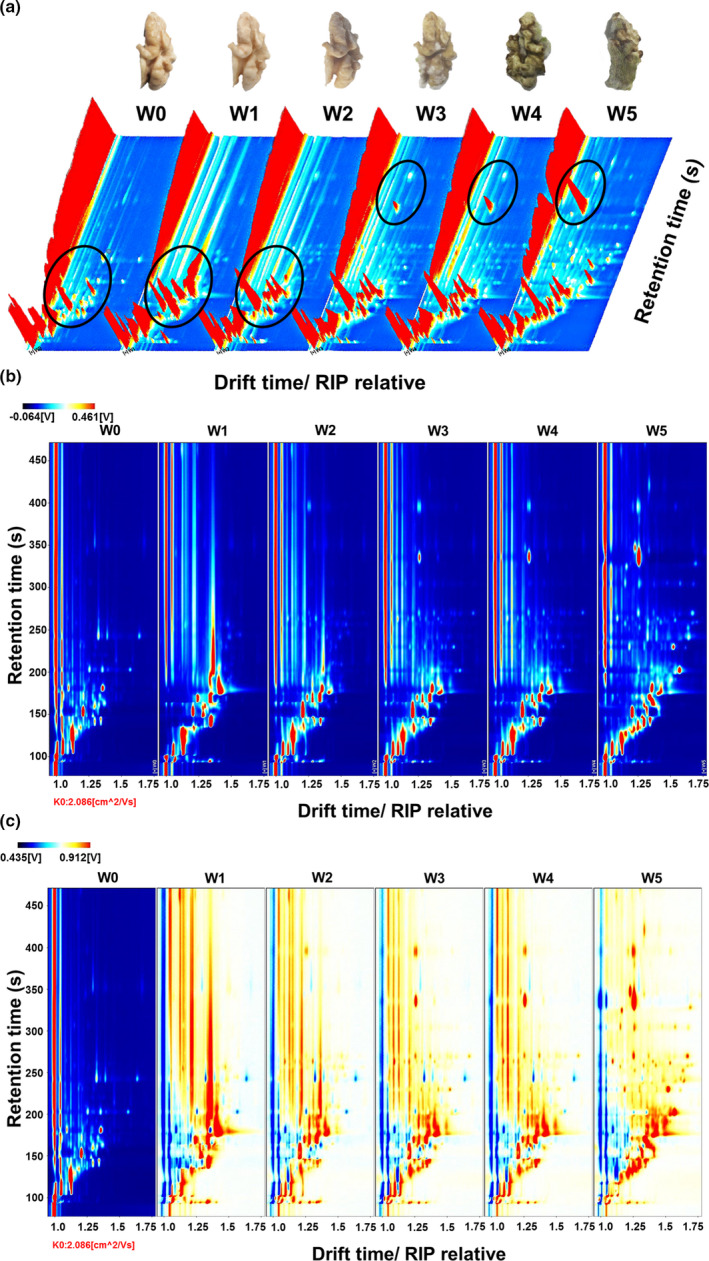
3d topographic and 2D topographic maps for walnut samples with different stages of mold growth. (a) The walnut samples and the 3D topographic plot of walnuts with different stages of mold growth; (b) the 2D topographic plot of walnuts at different times; and (c) the 2D difference spectrum plot of walnuts at different times. W0: walnut samples contaminated by *A. flavus* for 0 hr; W1: walnut samples contaminated by *A. flavus* for 12 hr; W2: walnut samples contaminated by *A. flavus* for 1 day; W3: walnut samples infected by *A. flavus* for 2 days; W4: walnut samples infected by *A. flavus* for 4 days; and W5: walnut samples infected by *A. flavus* for 6 days

The differences in volatile compounds in walnut samples with different degrees of *A. flavus* contamination as a function of time were analyzed by GC‐IMS. The data are represented by 3D topographical visualization in Figure 1a, where the y‐axis represents the retention time of the analysis in the gas chromatograph, the x‐axis represents the ion migration time for identification, and the z‐axis represents the peak height for quantification. As shown in Figure 1a, VOCs of walnut samples with different degrees of mold growth were very similar, but the signal intensity was slightly different. After infection by AFs, the contents of most flavor compounds decreased.

The ion migration time and the position of the reactive ion peak (RIP) were normalized. A top view of the GC‐IMS 3D topographic plot of walnut samples with different aflatoxin infection days is shown in Figure 1b and Figure 1c. The whole spectrum represented the total headspace compounds of the samples. Each point to the right of the RIP represents a volatile compound extracted from the samples. Most of the signals appeared in the retention time range of 100–900 s with a drift time of 1.0–1.5 s. The color represents the signal intensity of the compound. White indicates a lower intensity, and red indicates higher intensity. The darker the color, the greater the intensity.

The difference comparison model was applied to compare the differences between walnut samples. The topographic plot of uninfected walnut samples was selected as a reference, and the topographic plots of the other samples were normalized with the reference (Figure 1c). If the VOCs were consistent, the background after deduction was white, while red indicated that the concentration of the substance was higher than in the reference, and blue indicated that the concentration of the substance was lower than in the reference. Most of the signals in the topographic plot of the walnut samples appeared between the retention times of 100 and 450s, and in the infected walnut samples, there were several different signals. (The retention times were between 350 s and 450 s.) Moreover, the signal intensity was stronger than that observed in the pileus. This may be because the compounds yielding these signals were considered to be weakly polar, considering that nonpolar compounds have a longer retention time on nonpolar columns than polar compounds (Arroyo‐Manzanares et al., 2017). After being contaminated with *A. flavus*, the signals of some compounds (sensitive to temperature and easy to decompose or degrade) disappeared, or the signal intensity decreased (Figure 1c). In contrast, the enhanced intensity of some signals showed that the concentration of some compounds increased after contamination.

### 3.2 Volatile compound identification in walnut samples at different moldy growth stages

**FIGURE 2:**
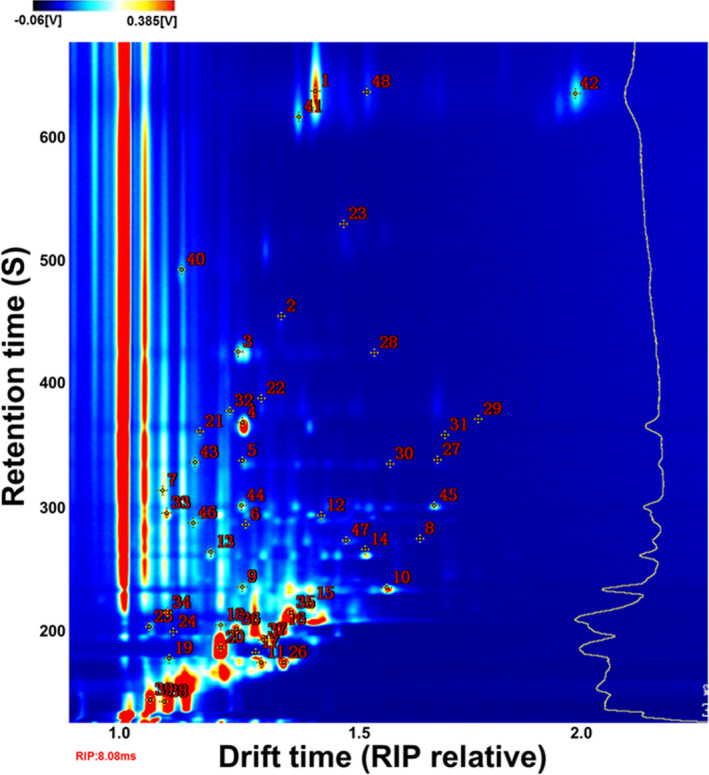
Ion migration spectra of walnuts infected by *A. flavus* for different periods of time. The numbers indicate identified VOCs

The compounds were characterized by comparing the IMS drift time and retention index with those of the authentic reference compounds. Due to their different concentrations, it was observed that some single compounds might produce multiple signals or spots (dimers or even trimers). A total of 48 typical compounds from the topographic plots were identified with a GC × IMS Library (Figure 2 and Table 1) and are represented by numbers in Figure 2. Furthermore, 15 typical compounds from the topographic plots were not identified as corresponding by names.

**TABLE 1:** The information on identified compounds of walnut samples

No.	Compound	CAS#	Formula	MW	RI	Rt [s]	Dt	Remarks
1	(E)−2‐nonenal‐M	18829–56–6	C_9_H_16_O	140.2	1,187.5	610.905	1.41366	Monomer
2	(E)−2‐octenal	2548–87–0	C_8_H_14_O	126.2	1,056.7	426.753	1.34043	Null
3	Benzeneacetaldehyde‐M	122–78–1	C_8_H_8_O	120.2	1,035.8	397.332	1.24744	Monomer
4	2‐pentyl furan	3777–69–3	C_9_H_14_O	138.2	993.1	339.092	1.25675	Null
5	(E)‐hept−2‐enal‐M	18829–55–5	C_7_H_12_O	112.2	955.6	307.807	1.257	Monomer
6	2‐heptanone	110–43–0	C_7_H_14_O	114.2	892.8	255.438	1.26259	Null
7	dihydro−2(3h)‐furanone	96–48–0	C_4_H_6_O_2_	86.1	926	283.099	1.08417	Null
8	*n*‐hexanol	111–27–3	C_6_H_14_O	102.2	869.7	243.494	1.63935	Null
9	Hexanal‐M	66–25–1	C_6_H_12_O	100.2	792.1	203.888	1.25566	Monomer
10	Hexanal‐D	66–25–1	C_6_H_12_O	100.2	791.4	203.574	1.56833	Dimer
11	V1	*	*	0	594.4	142.28	1.2981	Null
12	V2	*	*	0	901.5	262.668	1.42628	Null
13	2‐Hexen−1‐ol‐M	2305–21–7	C_6_H_12_O	100.2	848.8	232.807	1.18723	Monomer
14	2‐Hexen−1‐ol‐D	2305–21–7	C_6_H_12_O	100.2	852.5	234.693	1.52242	Dimer
15	V3	*	*	0	759	190.372	1.4029	Null
16	3‐hydroxybutan−2‐one‐D	513–86–0	C_4_H_8_O_2_	88.1	703.3	169.312	1.3414	Dimer
17	V4	*	*	0	630	150.389	1.28451	Null
18	V5	*	*	0	712.1	172.627	1.21012	Null
19	Ethyl acetate‐M	141–78–6	C_4_H_8_O_2_	88.1	607.1	145.172	1.09852	Monomer
20	V6	*	*	0	646.8	154.232	1.20866	Null
21	Oct−1‐en−3‐ol	3391–86–4	C_8_H_16_O	128.2	984	331.517	1.16386	Null
22	Hexanoic acid	142–62–1	C_6_H_12_O_2_	116.2	1,008.7	359.089	1.29704	Null
23	Nonanal	124–19–6	C_9_H_18_O	142.2	1,110.2	502.011	1.4751	Null
24	Propanoic acid	79–09–4	C_3_H_6_O_2_	74.1	697.9	167.287	1.10665	Null
25	3‐hydroxybutan−2‐one‐M	513–86–0	C_4_H_8_O_2_	88.1	708.6	171.344	1.05562	Monomer
26	Ethyl acetate‐D	141–78–6	C_4_H_8_O_2_	88.1	592.1	141.75	1.34555	Dimer
27	(E)‐hept−2‐enal‐D	18829–55–5	C_7_H_12_O	112.2	956.2	308.335	1.67703	Dimer
28	Benzeneacetaldehyde‐D	122–78–1	C_8_H_8_O	120.2	1,035.3	396.51	1.5406	Dimer
29	2‐Octanone	111–13–7	C_8_H_16_O	128.2	996.6	342.051	1.76611	Null
30	2‐Furanmethanol, 5‐methyl‐	3857–25–8	C_6_H_8_O_2_	112.1	952.3	305.035	1.57587	Null
31	V7	*	*	0	980.7	328.753	1.69394	Null
32	V8	*	*	0	1,001.3	348.712	1.22881	Null
33	3‐(methylthio)propanal	3268–49–3	C_4_H_8_OS	104.2	903.8	264.6	1.09279	Null
34	V9	*	*	0	739.7	183.069	1.09451	Null
35	V10	*	*	0	738.6	182.658	1.36158	Null
36	3‐methyl−2‐butanol	598–75–4	C_5_H_12_O	88.1	703.6	169.43	1.24442	Null
37	1,2‐dimethoxyethane	110–71–4	C_4_H_10_O_2_	90.1	674.9	160.638	1.30063	Null
38	V11	*	*	0	452	109.785	1.08838	Null
39	V12	*	*	0	458.8	111.345	1.05735	Null
40	Ortho‐guaiacol	90–05–1	C_7_H_8_O_2_	124.1	1,083.5	464.412	1.12538	Null
41	(E,Z)−2,6‐nonadienal	557–48–2	C_9_H_14_O	138.2	1,172.7	590.047	1.37729	Null
42	(E)−2‐nonenal‐D	18829–56–6	C_9_H_16_O	140.2	1,186.5	609.43	1.97516	Dimer
43	Benzaldehyde	100–52–7	C_7_H_6_O	106.1	953.9	306.369	1.15371	Null
44	V13	*	*	0	911.3	270.918	1.2549	Null
45	V14	*	*	0	911.1	270.683	1.67046	Null
46	Cyclohexanone	108–94–1	C_6_H_10_O	98.1	894.2	256.603	1.14968	Null
47	V15	*	*	0	866.9	242.059	1.4803	Null
48	1‐nonanol	143–08–8	C_9_H_20_O	144.3	1,187.3	610.577	1.5251	Null

Abbreviations: *, unidentified; Dt, drift time; MW, molecular mass; RI, retention index; Rt, retention time.

### 3.3 Changes in volatile compounds in walnut samples contaminated by A. flavus

**FIGURE 3:**
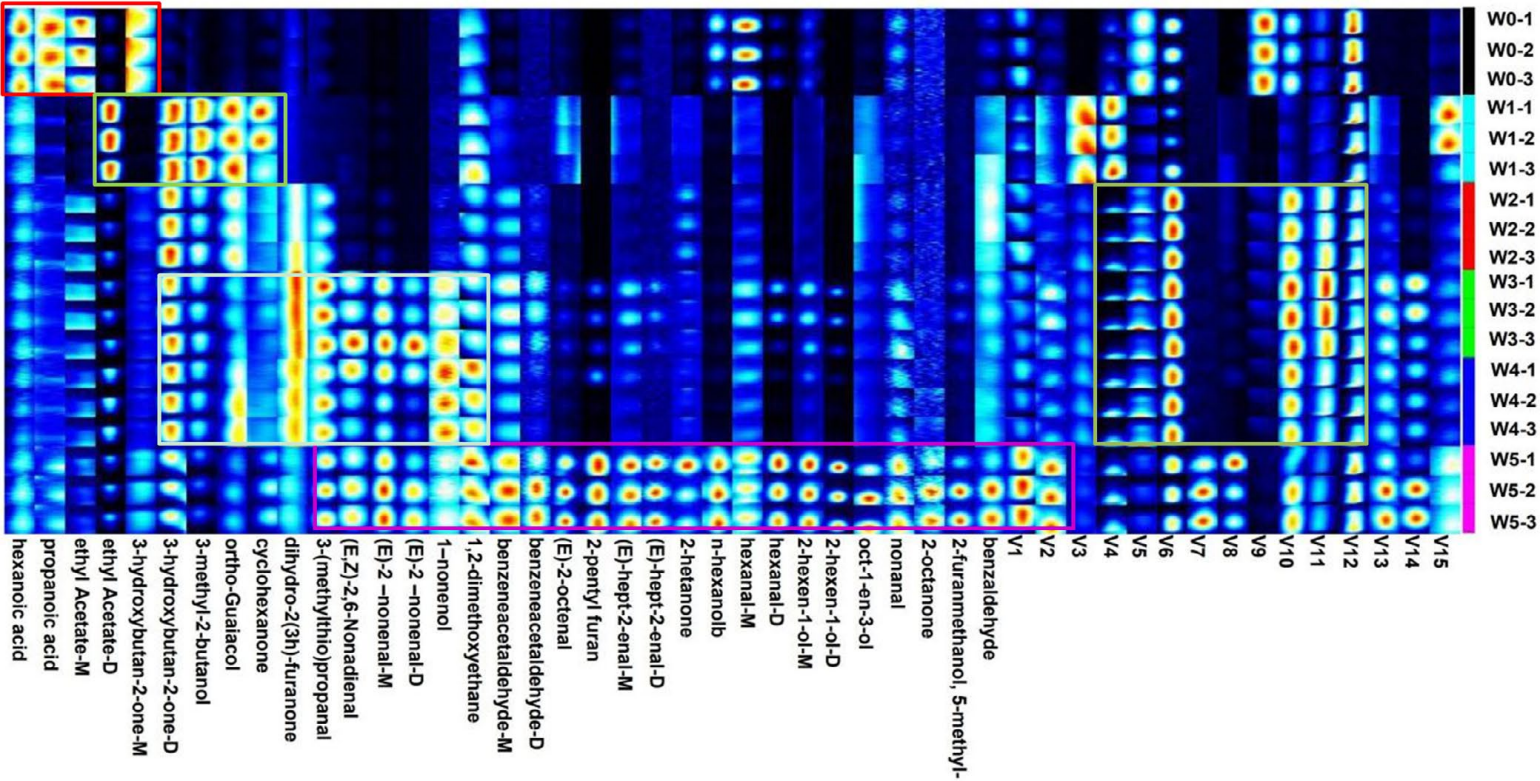
Fingerprint comparison of VOCs in noninoculated samples and *A. flavus* inoculated samples determined by HS‐GC‐IMS. Notes: The darker the spot, the larger is the quantity of volatile compounds. Each row represents all the signal peaks selected in a sample. Each column represents the signal peak of the same VOCs in different samples

The notable visual plots were chosen and listed together by gallery plot for intuitive comparison. Accordingly, the differences in volatile compounds in walnut samples with different contamination times were observed, and the characteristic fingerprints corresponding to each stage were established. As shown in Figure 3, the signals of hexanoic acid, propanoic acid, ethyl acetate‐M, and 3‐hydroxybutan‐2‐one‐M, V5, V9, and V12 in the W0 samples were much higher than those in the sample groups contaminated by *A. flavus*. Ethyl acetate‐D, 3‐hydroxybutan‐2‐one‐D, 3‐methyl‐2‐butanol, ortho‐guaiacol, and cyclohexanone were almost absent in the W0 samples. However, the signals of these volatile compounds were strongest in the W1 sample. Some volatile compounds, including (E, Z)‐2,6‐nonadienal, 1, 2‐dimethoxyethane, benzene acetaldehyde‐M, benzene acetaldehyde‐D, benzene acetaldehyde‐D, nonanal, (E)‐2‐octanone, 2‐pentyl furan, (E)‐hept‐2‐enal‐M, (E)‐hept‐2‐enal‐D, (E)‐2‐nonenal‐D, (E)‐2‐nonenal‐M, 1‐nonanol, 1,2‐dimethoxyethane, 2‐heptanone, n‐hexanol, hexanal‐D, 2‐hexen‐1‐ol‐m, oct‐1‐en‐3‐ol, 2‐hexen‐1‐0l‐D, oct‐1‐en‐3‐ol, nonanal, 2‐octanone, 2‐furanmethanol, benzaldehyde, V1, V2, V7, V8, V13, and V14 in the W5 sample group, were much higher than those in the W0 sample group.

On the other hand, the signal of 1, 2‐dimethoxyethane, benzene acetaldehyde‐M, benzene acetaldehyde‐D, (E)‐hept‐2‐enal‐M, 2‐heptanone, oct‐1‐en‐3‐ol, nonanal, 2‐octanone, and benzaldehyde increased with increasing *A. flavus* contamination time. In addition, walnut samples in the W5 group had more unique flavor compounds and higher volatile compound concentrations than those in the W0 group, and the volatile compounds identified in the stipe were more abundant. At the same time, there were fewer unique flavor compounds in the walnut sample that were not contaminated by *A. flavus*.

**FIGURE 4:**
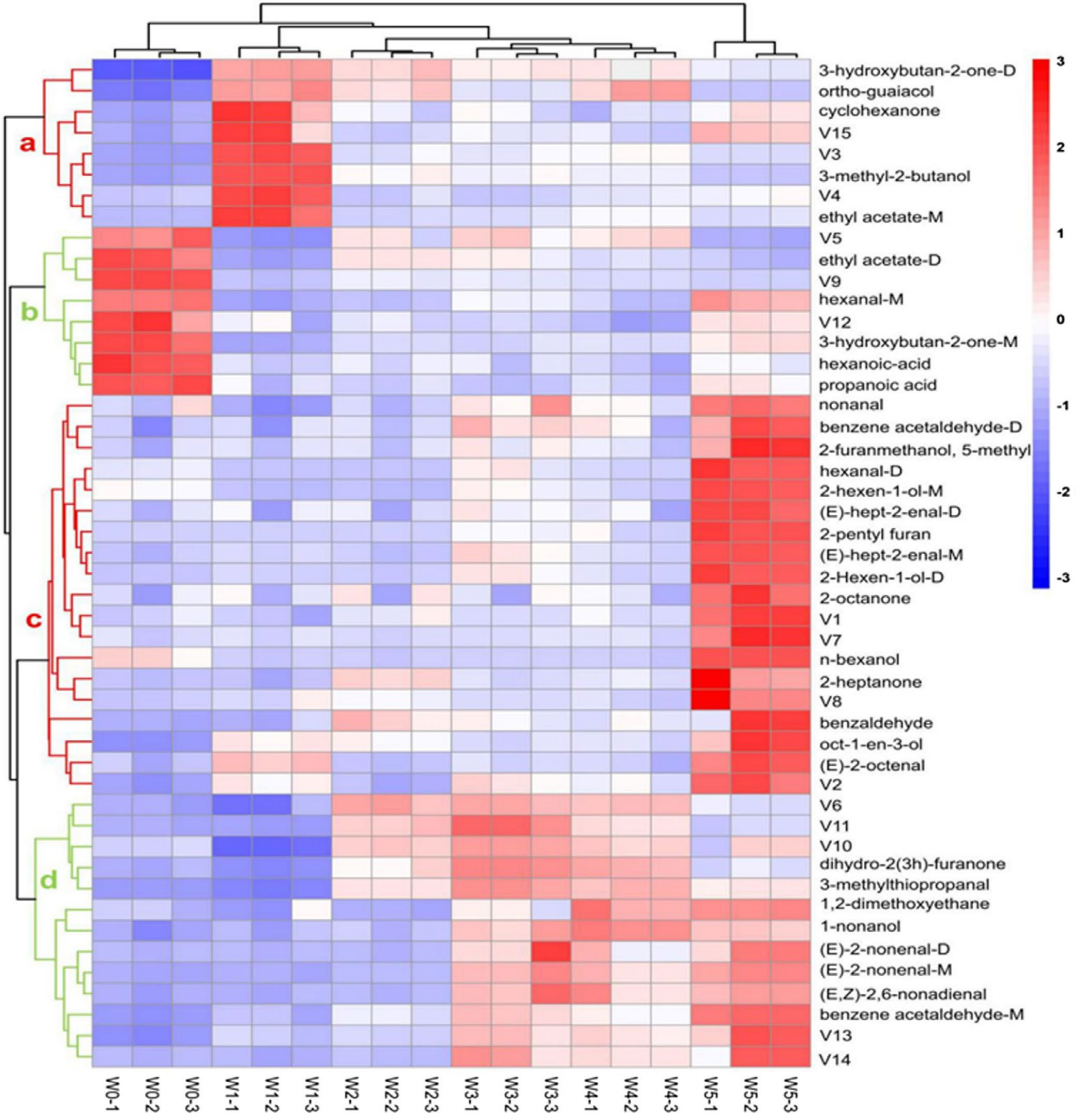
Heat map and cluster analysis of walnut samples with different extents of infection time

To further understand the differences in VOCs of walnut samples contaminated by *A. flavus* in different mold growth stages, cluster analysis was performed using a heat map (Figure 4). According to the vertical direction of the heat map, all samples were classified into four main categories: the control group, early‐stage mold, midstage mold, and late‐stage mold. The volatile compounds in walnut samples could be divided into four groups: clusters a, b, c, and d. At the midstage mold period of *A. flavus* contamination, the volatile components of V2‐V4 were similar to those of W0 and W1. Moreover, they were quite different from those in the late‐stage mold group. In addition, volatile compounds of group b were mainly present in W0, that of groups b and d were mainly produced in the early‐stage mold samples and midstage mold samples, while that of group c were mainly produced during the late‐stage mold samples. As is shown in Figure 4, there were many kinds of volatile compounds in the walnut samples, and the signals of these volatile compounds were higher in the late‐stage mold samples than in the samples from other periods. However, a significant amount of *A. flavus* could be seen in the late‐stage mold samples (Figure 1a). Therefore, in order to achieve early monitoring and warning, we focused on the detection targets of these compounds in groups a and d (Figure 4). The compounds in group a ethyl acetate‐D, 3‐methyl‐2‐butanol, cyclohexanone, V3, V4, and V15 were strongest in the premold stages, and it can be seen that the signals weakened as growth time increased.

### 3.4 Similarity analysis of fingerprints based on PCA

**FIGURE 5:**
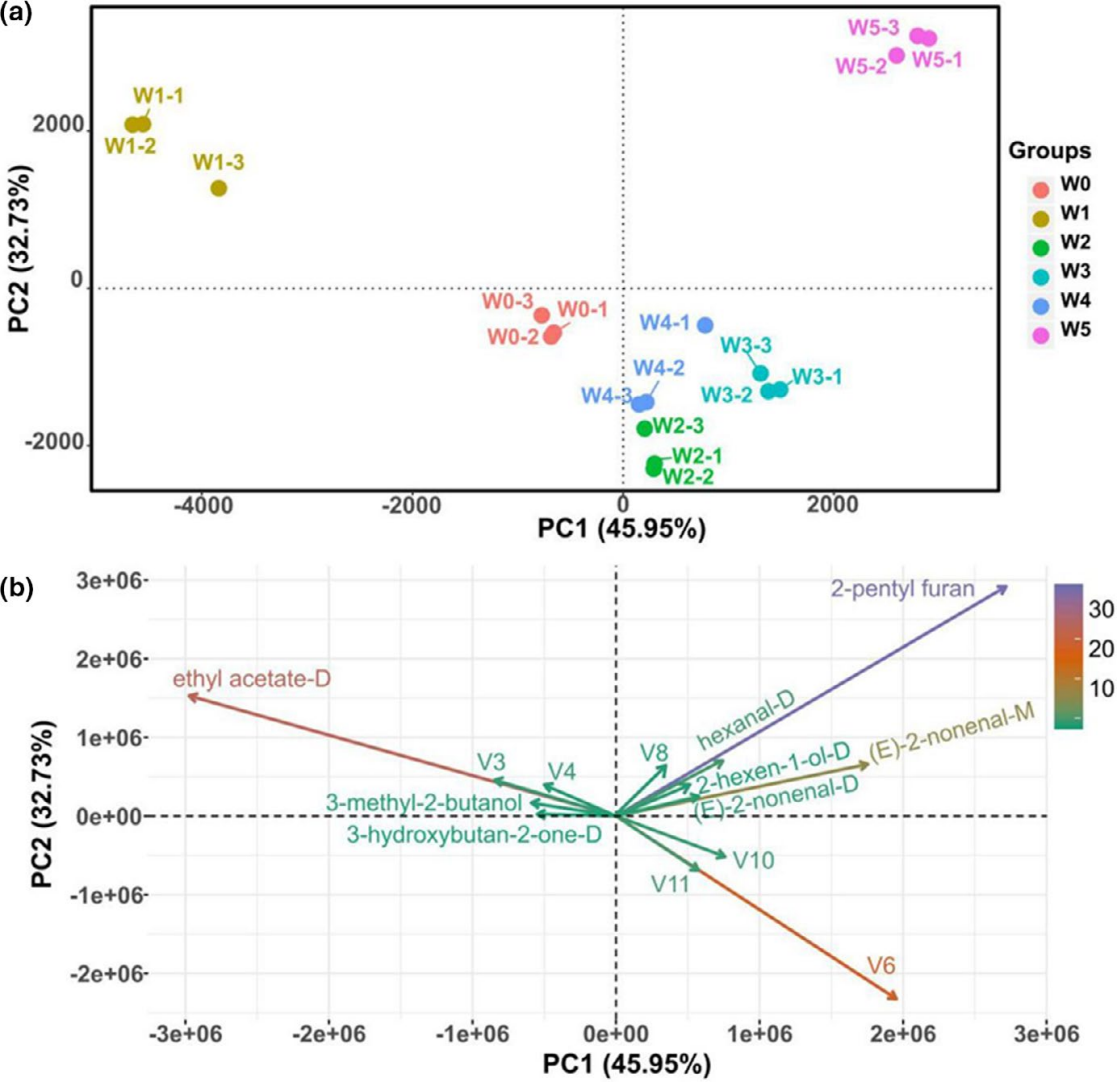
Principal component analysis based on the signal intensity obtained from walnut samples

Principal component analysis is a multivariate statistical analysis technique that uses multiple variables to linearly transform data to select a few significant variables (Yang et al., 2019). By determining a few principal component factors to represent many complex and difficult‐to‐find variables in the original samples, the regularity and difference among samples could be evaluated according to the contribution rate of principal component factors in different samples (Sebzalli & Wang, 2001). PCA was established using signal intensity to highlight the differences in volatile compounds. The PCA of volatile compounds in walnuts with different extents of *A. flavus* contamination is shown in Figure 5a. The PCA results clearly show that the PCA biplots PC1 and PC2 accounted for 78.68% of the total variance in the dataset (Figure 5a). The distribution map for the first two principal components determined by PCA is shown in the figure, and PC1 and PC2 described 45.95% and 32.73% of the accumulative variance, respectively. These components were thought to exhibit the similarity between the different walnut samples.

As shown in Figure 5a, the control group and treatment group walnut samples were in a relatively independent space and well‐distinguished in the distribution map. It was evident that four distinct regions could be identified within the PCA profile (Figure 5a). The treatment group walnut samples could be well‐defined according to the positive score values of PC1 and PC2. Among them, the samples of groups V2, V3, and V4 were relatively close, which indicated that the volatile components of these samples were similar. There were significant differences between them and the V1 and V5 samples. Based on the PCA results, the *A. flavus* infection at different stages of mold growth on the walnut samples was well‐separated. To obtain more details, the biplots were used (Figure 5b). As shown in Figure 5b, the direction and length of the vector indicated the contribution of the variables toward the two principal components. According to the above results, we could infer that the ethyl acetate‐D was positively related to the control group of walnut samples. However, for the late‐stage mold samples, 2‐pentyl furan contributed greatly to the flavor profile. When walnut samples were subjected to midstage of *A. flavus* growth, V1, V10, and V6 were positively correlated with the walnut samples. In the biplots, the relationship between specific volatile components and walnut samples at different contamination stages was demonstrated. This finding was consistent with the fingerprints and heat maps.

## 4 DISCUSSION

Today, global walnut production is increasing because of the increasing consumer demand for this food. The global production of walnuts is approximately 1,500,000 metric tons, and China, the United States, and Iran are the major producers of walnuts (Amini & Ghoranneviss, 2016). However, walnuts are susceptible to infection during storage, and their deterioration occurs due to the *A. flavus* activity (Golge et al., 2016). Therefore, monitoring and controlling *Aspergillus flavus*‐based quality during walnut storage is one of the main objectives of proper walnut storage. Moreover, rapid and nondestructive detection of walnut quality based on *A. flavus* is also a critical issue for microbiologists, the walnut industry, and those monitoring the quality and safety of walnuts. However, rapid and accurate early warning methods for minimizing mold damage are still lacking, especially a type of fast and no pretreatment, nondestructive testing detection method, or real‐time online monitoring. In a word, identifying and determining the degree of *A. flavus* contamination in walnuts are necessary for the development of preservation techniques, and rapid and early detection of *A. flavus* in walnuts is also critical at every stage of storage and processing. HS‐GC‐IMS showed potential for evaluating walnut volatile composition over time with high‐throughput capabilities. In recent years, HS‐GC‐IMS has been extensively used not only in the investigation of volatile compounds in food science (Hernández‐Mesa et al., 2019), but also in the identification of human pathogens (Jünger et al., 2008). This technique implements a convenient and efficient instrument with the advantages of simple sample preparation, easy operation, high sensitivity, and quick analytical speed. Even trace volatile compounds can also be detected in a short time with this technique (Li et al., 2019). As a consequence, HS‐GC‐IMS could be used to identify the volatile components of walnuts with different stages of *A. flavus* contamination. These results provided valid targets for the development of sensors to evaluate the early mold contamination in stored walnut. And, based on the objectives identified in this study, it is helpful for us to propose an online monitoring and early warning system model, which is also the later research direction.

A total of 48 signal peaks from topographic plots were identified in walnut samples under different *A. flavus* contamination states in this study. The volatile components produced by fungal respiration were analyzed qualitatively and quantitatively to distinguish walnuts in the different contamination states. In addition, the results of PCA also clearly showed that the different samples were in relatively independent spaces and were well‐distinguished. After the volatile compounds were identified and multivariate data analysis was conducted, the potential biomarkers in different stages of *A. flavus* contamination of walnuts were highlighted. The compounds ethyl acetate‐D, 3‐methyl‐2‐butanol, cyclohexanone, V3, V4, and V15 were strongest in the premold stages, and it can be seen that the signals weakened as growth time increased. These results indicated that it is possible to feasible to establish a suitable gas sensor to monitor early mold formation in stored walnuts.

In this study, a simple, specific, and reliable method was developed to evaluate the characteristic volatile compounds of walnut samples contaminated by *A. flavus* by establishing their unique compound fingerprints with HS‐GC‐IMS and PCA, which required minimal sample preparation steps and reduced the time required for analysis. Given its advantages, HS‐GC‐IMS fingerprint coupled with PCA could be used to identify the degree of *A. flavus* contamination in walnuts. This study provides a new insight into monitoring the *A. flavus* contamination levels in walnuts. Besides, the most important thing is early monitoring of *A. flavus* contamination and is of great significance for global food security.

## CONFLICT OF INTEREST

The authors declare no conflict of interest.

## ETHICAL APPROVAL

This study does not involve any human or animal testing.
